# Telework Before Illness Onset Among Symptomatic Adults Aged ≥18 Years With and Without COVID-19 in 11 Outpatient Health Care Facilities — United States, July 2020

**DOI:** 10.15585/mmwr.mm6944a4

**Published:** 2020-11-06

**Authors:** Kiva A. Fisher, Samantha M. Olson, Mark W. Tenforde, Leora R. Feldstein, Christopher J. Lindsell, Nathan I. Shapiro, D. Clark Files, Kevin W. Gibbs, Heidi L. Erickson, Matthew E. Prekker, Jay S. Steingrub, Matthew C. Exline, Daniel J. Henning, Jennifer G. Wilson, Samuel M. Brown, Ithan D. Peltan, Todd W. Rice, David N. Hager, Adit A. Ginde, H. Keipp Talbot, Jonathan D. Casey, Carlos G. Grijalva, Brendan Flannery, Manish M. Patel, Wesley H. Self, IVY Network Investigators, CDC COVID-19 Response Team, IVY Network Investigators, Kimberly W. Hart, Robert McClellan, Hsi-nien Tan, Adrienne Baughman, CDC COVID-19 Response Team, Nora A. Hennesy, Brittany Grear, Michael Wu, Kristin Mlynarczyk, Luc Marzano, Zuwena Plata, Alexis Caplan, E. Ogokeh, Emily R. Smith, Sara S. Kim, Eric P. Griggs, Bridget Richards, Sonya Robinson, Kaylee Kim, Ahmed M. Kassem, Courtney N. Sciarratta, Paula L. Marcet

**Affiliations:** ^1^CDC COVID-19 Response Team; ^2^Epidemic Intelligence Service, CDC; ^3^Influenza Vaccine Effectiveness in the Critically Ill (IVY) Network; ^4^Vanderbilt University Medical Center, Nashville, Tennessee; ^5^Beth Israel Deaconess Medical Center, Boston, Massachusetts; ^6^Wake Forest University Baptist Medical Center, Winston-Salem, North Carolina; ^7^Hennepin County Medical Center, Minneapolis, Minnesota; ^8^Baystate Medical Center, Springfield, Massachusetts; ^9^Ohio State University Wexner Medical Center, Columbus, Ohio; ^10^University of Washington Medical Center, Seattle, Washington; ^11^Stanford University Medical Center, Palo Alto, California; ^12^Intermountain Healthcare, Salt Lake City, Utah; ^13^Johns Hopkins Hospital, Baltimore, Maryland; ^14^University of Colorado School of Medicine, Aurora, Colorado.; Vanderbilt University Medical Center; Vanderbilt University Medical Center; Vanderbilt University Medical Center; Vanderbilt University Medical Center.; CDC COVID-19 Response Team; CDC COVID-19 Response Team; CDC COVID-19 Response Team; CDC COVID-19 Response Team; CDC COVID-19 Response Team; CDC COVID-19 Response Team; CDC COVID-19 Response Team; CDC COVID-19 Response Team; CDC COVID-19 Response Team; CDC COVID-19 Response Team; CDC COVID-19 Response Team; CDC COVID-19 Response Team; CDC COVID-19 Response Team; CDC COVID-19 Response Team; CDC COVID-19 Response Team; CDC COVID-19 Response Team; CDC COVID-19 Response Team.

Since March 2020, large-scale efforts to reduce transmission of SARS-CoV-2, the virus that causes coronavirus disease 2019 (COVID-19), have continued. Mitigation measures to reduce workplace exposures have included work site policies to support flexible work site options, including telework, whereby employees work remotely without commuting to a central place of work.* Opportunities to telework have varied across industries among U.S. jobs where telework options are feasible (*1*). However, little is known about the impact of telework on risk for SARS-CoV-2 infection. A case-control investigation was conducted to compare telework between eligible symptomatic persons who received positive SARS-CoV-2 reverse transcription–polymerase chain reaction (RT-PCR) test results (case-patients, 153) and symptomatic persons with negative test results (control-participants, 161). Eligible participants were identified in outpatient health care facilities during July 2020. Among employed participants who reported on their telework status during the 2 weeks preceding illness onset (248), the percentage who were able to telework on a full- or part-time basis was lower among case-patients (35%; 42 of 120) than among control-participants (53%; 68 of 128) (p<0.01). Case-patients were more likely than were control-participants to have reported going exclusively to an office or school setting (adjusted odds ratio [aOR] = 1.8; 95% confidence interval [CI] = 1.2–2.7) in the 2 weeks before illness onset. The association was also observed when further restricting to the 175 participants who reported working in a profession outside the critical infrastructure^†^ (aOR = 2.1; 95% CI = 1.3–3.6). Providing the option to work from home or telework when possible, is an important consideration for reducing the risk for SARS-CoV-2 infection. In industries where telework options are not available, worker safety measures should continue to be scaled up to reduce possible worksite exposures.

This multistate case-control study assessed possible exposures to COVID-19. Methods have been described elsewhere (*2*). In brief, the investigation included symptomatic adults aged ≥18 years who received their first SARS-CoV-2 test at one of 11 Influenza Vaccine Effectiveness in the Critically Ill (IVY) Network outpatient testing or health care centers^§^ during July 1–29, 2020 (*3*). Laboratory-confirmed case-patients were randomly sampled. Two control-participants were matched based on age, sex, and study location to each case patient, resulting in 615 potential case-patients and 1,212 control-participants. Case-patients and control-participants were contacted 14–23 days after their SARS-CoV-2 test and interviewed to identify participants who were symptomatic and had not been previously tested for SARS-CoV-2. A total of 802 adults (295 case-patients and 507 control-participants) agreed to participate in structured interviews in English or five other languages^¶^ administered by CDC personnel via telephone with data collected in REDCap software (version 10.3.8; REDCap Consortium) (*4*); 163 adults (9%) declined to participate.

Among these 802 adults contacted, 470 (59%) were ineligible (i.e., were not symptomatic or had a previous SARS-CoV-2 test), and 18 (2%) were excluded because of nonresponse to the telework and work-from-home question. The final analytic sample (314) included 153 (49%) case-patients and 161 (51%) control-participants. An unmatched analysis was performed because of the strict inclusion criteria that resulted in many participants being ineligible for the investigation. This activity was reviewed by CDC and participating sites and conducted consistent with applicable federal law and CDC policy.**

Data collected in this investigation included self-reported demographic characteristics, underlying chronic medical conditions,^††^ employment status and location, telework status, close contact (within 6 feet for ≥15 minutes) with a person with known COVID-19, and community exposures (*2*). All questions relating to employment, close contact, and community exposures were asked with reference to the 14 days preceding illness onset. Participants who reported working full-time, part-time, who were self-employed, or who were students were asked additional questions about work, including type of work and telework status.^§§^ Descriptive and statistical analyses were performed to assess differences between case-patients and control-participants, as well as between those who reported teleworking and going to an office setting, in terms of demographic characteristics, possible workplace, close contact, and community exposures.

Unconditional logistic regression models, accounting for site-level clustering, were used to estimate odds ratios and 95% CIs for associations between telework status and case-patients and control-participants, adjusting for age, sex, race/ethnicity, and presence of one or more underlying chronic medical conditions (*2*). Analysis was conducted for all participants who reported work and telework status (248) and was then restricted to those working outside the selected critical infrastructure sectors measured (i.e., not in health care, factory, corrections, or education settings) (175). Significance levels were set at p<0.05. Statistical analyses were conducted using SAS software (version 9.4; SAS Institute).

Compared with case-patients, control-participants were more likely to be non-Hispanic White (p<0.01), have a college degree or higher (p<0.01), and report at least one underlying chronic medical condition (p = 0.02) (Table 1). In the 14 days before illness onset, 59% of case-patients and 68% of control-participants reported working full-time. Among the 262 participants who reported some form of employment, 36 (30%) case-patients and 37 (27%) control-participants reported workplace or school closures. Three quarters of case-patients (75%) and nearly two thirds of control-participants (66%) worked outside the critical infrastructure. Just over a third of case-patients (35%) reported working from home or teleworking at least part of the time, compared with approximately one half (53%) of control-participants (p<0.01).

**TABLE 1:** Characteristics of symptomatic adults aged ≥18 years who were outpatients in 11 academic health care facilities and who received positive and negative SARS-CoV-2 test results (314)* — United States, July 1–29, 2020

Characteristic	No. (%)		p-value
	Case-patients (153)	Control-participants (161)	
**Age group, yrs**			
18–29	44 (28.7)	39 (24.2)	0.23
30–44	46 (30.1)	62 (38.5)	
45–59	45 (29.4)	36 (22.4)	
≥60	18 (11.8)	24 (14.9)	
**Sex**			
Men	75 (49.0)	72 (44.7)	0.45
Women	78 (51.0)	89 (55.3)	
**Race/Ethnicity (missing = 1)**			
White, non-Hispanic	92 (60.5)	124 (77.0)	<0.01
Hispanic/Latino	29 (19.1)	12 (7.5)	
Black, non-Hispanic	25 (16.5)	19 (11.8)	
Other, non-Hispanic**^†^**	6 (3.9)	6 (3.7)	
**Education (missing = 4)**			
Less than high school	15 (9.9)	3 (1.9)	<0.01
High school degree or some college	60 (39.5)	48 (30.4)	
College degree or more	77 (50.6)	107 (67.7)	
**Health insurance coverage (missing = 2)** ^§^			
No insurance	15 (9.8)	9 (5.7)	0.16
Yes	130 (85.0)	146 (91.8)	
Don’t know	8 (5.2)	4 (2.5)	
**At least one underlying chronic medical condition**^¶^ **(missing = 1)**	74 (48.4)	98 (61.3)	0.02
**Type of residence (missing = 1)**			
Single family home	107 (69.9)	119 (74.4)	0.41
Apartment	34 (22.2)	34 (21.2)	
Other**	12 (7.9)	7 (4.4)	
**Household income (US$)**			
<25,000	20 (13.1)	10 (6.2)	0.09
25,000–34,000	10 (6.5)	8 (5.0)	
35,000–49,000	16 (10.5)	12 (7.5)	
50,000–74,000	17 (11.1)	25 (15.5)	
≥75,000	64 (41.8)	87 (54.0)	
Don't know/Not sure	15 (9.8)	8 (5.0)	
Refused	11 (7.2)	11 (6.8)	
**Employment status 14 days before illness onset**			
Work full-time	90 (58.8)	109 (67.7)	0.45
Work part-time	23 (15.0)	18 (11.2)	
Self-employed	8 (5.2)	6 (3.7)	
Student	6 (3.9)	2 (1.2)	
Homemaker	5 (3.3)	4 (2.5)	
Retired	10 (6.6)	14 (8.7)	
Not employed currently/Unable to work	11 (7.2)	8 (5.0)	
**Workplace or school closure because of COVID-19 during illness (256)**	36 (29.8)	37 (27.4)	0.68
**Place of employment 14 days before illness onset (262)**			
Health care facility (not in a long-term care facility)	19 (15.0)	28 (20.8)	0.44
Health care facility (long-term care facility)	1 (0.8)	3 (2.2)	
Large factory setting	4 (3.1)	5 (3.7)	
Correctional or detention facility	0 (0.0)	2 (1.5)	
Teacher, educator, or camp counselor^††^	8 (6.3)	8 (5.9)	
Other^§§^	95 (74.8)	89 (65.9)	
**Telework and office or school attendance 14 days before illness onset (248)** ^¶¶^			
Worked from home or teleworked at least part of the time	42 (35.0)	68 (53.1)	<0.01
Went into an office or school regularly	78 (65.0)	60 (46.9)	

* Patients were randomly sampled from 11 academic health care systems that are part of the Influenza Vaccine Effectiveness in the Critically Ill (IVY) Network sites (Baystate Medical Center, Springfield, Massachusetts; Beth Israel Deaconess Medical Center, Boston, Massachusetts; University of Colorado School of Medicine, Aurora, Colorado; Hennepin County Medical Center, Minneapolis, Minnesota; Intermountain Healthcare, Salt Lake City, Utah; Ohio State University Wexner Medical Center, Columbus, Ohio; Wake Forest University Baptist Medical Center, Winston-Salem, North Carolina; Vanderbilt University Medical Center, Nashville, Tennessee; Johns Hopkins Hospital, Baltimore, Maryland; Stanford University Medical Center, Palo Alto, California; University of Washington Medical Center, Seattle, Washington). Participating states include California, Colorado, Maryland, Massachusetts, Minnesota, North Carolina, Ohio, Tennessee, Utah, and Washington.

^†^ Other race includes responses of Native American/Alaska Native, Asian, Native Hawaiian/other Pacific Islander, and other; these were combined because of small sample sizes.

^§^ Insurance status included public, private, or both. No insurance included those who reported having neither private nor public insurance.

^¶^ Reported at least one of the following underlying chronic medical conditions: cardiac condition, hypertension, asthma, chronic obstructive pulmonary disease, immunodeficiency, psychiatric condition, diabetes, or obesity.

** Other residence included not specified or refused to answer (5), duplex/two-family home (3), trailer/mobile home (3), group home (2), townhome (2), hotel (1), long-term care facility (1), condominium (1), and lived in university fraternity or sorority housing (1).

^††^ Including any other field that works with children aged <18 years.

^§§^ Other work exposures are those who reported “No, I do not work in any of these fields” among the possible workplace exposures assessed.

^¶¶^ Thirteen participants reported “don’t know/not sure,” and one refused to answer the question. Participants were asked “In the 14 days prior to becoming ill, were you: Going into an office/school regularly; Working from home/teleworking; Both.” Response options were dichotomized with those who reported “both” categorized as “Worked from home or teleworked at least part of the time.”

A total of 110 (35%) participants reported teleworking or working from home at least part of the time, and 138 (44%) reported going into an office or school regularly 2 weeks before illness onset (Table 2). Participants who reported teleworking were more likely to be non-Hispanic White (p<0.01), have a college degree or higher (p<0.01), have health insurance (p<0.01), an income of ≥$75,000 (p<0.01), and report close contact with a person with a known COVID-19 case (p = 0.03). No significant differences were noted in most community exposures, including shopping, going to a salon, gym, restaurant, or bar/coffee shop or using public transportation, between participants who did and did not report teleworking. However, those who regularly attended work or school were also more likely to attend church or religious gatherings (15; 11%), compared with those who teleworked at least part of the time (three; 3%) (p = 0.01).

**TABLE 2:** Characteristics of work activity among symptomatic adults aged ≥18 years who reported working in the 14 days before illness onset from 11 academic health care facilities (248) * — United States, July 1–29, 2020

Characteristic	No. (%)		p-value
	Telework and work from home (110)	Going into an office or school regularly (138)	
**Age group, yrs**			
18–29	30 (27.3)	44 (31.9)	0.89
30–44	42 (38.2)	49 (35.5)	
45–59	31 (28.2)	37 (26.8)	
≥60	7 (6.3)	8 (5.8)	
**Sex**			
Men	48 (43.6)	71 (51.5)	0.22
Women	62 (56.4)	67 (48.5)	
**Race/Ethnicity (missing = 1)**			
White, non-Hispanic	87 (79.8)	84 (60.9)	<0.01
Hispanic/Latino	6 (5.5)	27 (19.6)	
Black, non-Hispanic	11 (10.1)	22 (15.9)	
Other, non-Hispanic^†^	5 (4.6)	5 (3.6)	
**Education (missing = 3)**			
Less than high school	1 (0.9)	9 (6.6)	<0.01
High school degree or some college	18 (16.7)	65 (47.4)	
College degree or more	89 (82.4)	63 (46.0)	
**Health insurance coverage (missing = 2)** ^§^			
No insurance	2 (1.8)	17 (12.4)	<0.01
Yes	104 (95.4)	114 (83.2)	
Don’t know	3 (2.8)	6 (4.4)	
**Household income (US$)**			
<25,000	4 (3.6)	18 (13.0)	<0.01
25,000–34,000	5 (4.6)	8 (5.8)	
35,000–49,000	3 (2.7)	16 (11.6)	
50,000–74,000	17 (15.5)	18 (13.0)	
≥75,000	69 (62.7)	64 (46.4)	
Don't know/Not sure	4 (3.6)	9 (6.5)	
Refused	8 (7.3)	5 (3.7)	
**Employment status 14 days before illness onset**			
Work full-time	85 (77.3)	107 (77.5)	0.12
Work part-time	12 (10.9)	24 (17.4)	
Self-employed	7 (6.4)	5 (3.6)	
Student	6 (5.4)	2 (1.5)	
**Place of employment 14 days before illness onset**			
Health care facility (not in a long-term care facility)	12 (10.9)	34 (24.6)	<0.01
Health care facility (long-term care facility)	1 (0.9)	3 (2.2)	
Large factory setting	0 (0.0)	6 (4.4)	
Correctional or detention facility	2 (1.8)	0 (0.0)	
Teacher, educator, or camp counselor^¶^	10 (9.1)	5 (3.6)	
Other**	85 (77.3)	90 (65.2)	
**Close contact with a person with known COVID-19 (missing = 2)**	26 (23.6)	50 (36.8)	0.03
**Community exposure 14 days before illness onset** ^††^			
Shopping (missing = 2)	100 (90.9)	119 (87.5)	0.40
Home, ≤10 persons (missing = 1)	66 (60.0)	68 (49.6)	0.10
Restaurant (missing = 2)	34 (30.9)	51 (37.5)	0.28
Salon (missing = 2)	21 (19.1)	17 (12.5)	0.16
Home, >10 persons (missing = 1)	19 (17.3)	16 (11.7)	0.21
Gym (missing = 2)	13 (11.8)	7 (5.2)	0.06
Public transportation (missing = 2)	5 (4.6)	8 (5.9)	0.64
Bar/Coffee shop (missing = 3)	7 (6.4)	13 (9.6)	0.35
Church/Religious gathering (missing = 2)	3 (2.7)	15 (11.0)	0.01

* Participants were asked “In the 14 days prior to becoming ill, were you: Going into an office/school regularly; Working from home/teleworking; Both.” Among 262 participants who reported working in the 14 days before illness onset, 13 reported “don’t know/not sure,” and one refused to answer the question. Response options were dichotomized with those who reported “both” as teleworking or working from home at least part of the time. Patients were randomly sampled from 11 academic health care systems that are part of the Influenza Vaccine Effectiveness in the Critically Ill (IVY) Network sites (Baystate Medical Center, Springfield, Massachusetts; Beth Israel Deaconess Medical Center, Boston, Massachusetts; University of Colorado School of Medicine, Aurora, Colorado; Hennepin County Medical Center, Minneapolis, Minnesota; Intermountain Healthcare, Salt Lake City, Utah; Ohio State University Wexner Medical Center, Columbus, Ohio; Wake Forest University Baptist Medical Center, Winston-Salem, North Carolina; Vanderbilt University Medical Center, Nashville, Tennessee; Johns Hopkins Hospital, Baltimore, Maryland; Stanford University Medical Center, Palo Alto, California; University of Washington Medical Center, Seattle, Washington). Participating states include California, Colorado, Maryland, Massachusetts, Minnesota, North Carolina, Ohio, Tennessee, Utah, and Washington.

^†^ Other race includes responses of Native American/Alaska Native, Asian, Native Hawaiian/other Pacific Islander, and other; these were combined because of small sample sizes.

^§^ Insurance status included public, private, or both. No insurance included those who reported having neither private nor public insurance.

^¶^ Including any other field that works with children aged <18 years.

** Persons who reported “No, I do not work in any of these fields” among the possible workplace exposures assessed.

^††^ Participants were asked “In the 14 days before feeling ill about how often did you: 1) Shop for items (groceries, prescriptions, home goods, clothing, etc.); 2) have people visit you inside your home or go inside someone else's home where there were more than 10 people; 3) have people visit you inside your home or go inside someone else's home where there were 10 people or less; 4) go to a restaurant (dine-in, any area designated by the restaurant including patio seating); 5) go to a gym or fitness center; 6) go to a salon or barber (e.g., hair salon, nail salon, etc.); 7) attend church or a religious gathering/place of worship; 8) go to a bar or coffee shop (indoors); and 9) use public transportation (bus, subway, streetcar, train, etc.).” Response options were coded as never versus at least once in the 14 days before illness onset. Participants were asked each question separately and could have responded to multiple community exposure questions.

Among the 248 participants who reported telework status and some form of employment during the 2 weeks before illness onset, case-patients were more likely to have reported exclusively going to an office or school setting (aOR = 1.8, 95% CI = 1.2–2.7) in the 2 weeks before illness onset than were control-participants. The association persisted when the analysis was restricted to the 175 participants who reported working in a profession outside the selected critical infrastructure sectors (aOR = 2.1, 95% CI = 1.3–3.6).

## Discussion

This investigation provides evidence of the potential health benefits of teleworking associated with the COVID-19 pandemic. Among participants who reported being employed during the 2 weeks preceding illness onset, the percentage who reported teleworking on a full- or part-time basis was significantly lower among case-patients (35%) than among control-participants (53%). For case-patients and control-participants, the percentage who reported teleworking is higher than national estimates that suggest 26% of U.S. adults were teleworking because of COVID-19 during July 2020 (*5*). Compared with control-participants, case-patients had higher odds of reporting regularly attending work or school. The association persisted when restricting the analysis to those who do not represent critical infrastructure workers measured in the survey. However, these findings highlight socioeconomic differences among participants who did and did not report teleworking before illness onset, with non-White employees and those who earn less money having less opportunity to telework. Sociocultural disparities and unemployment have also been observed in industries where telework options are not feasible (*5*–*7*).

Most community exposures were not associated with teleworking. Further studies are needed to better characterize the constellation of activities, including possible work and community exposures concomitantly occurring that could increase risk for infection, particularly while asymptomatic transmission occurs.

The findings in this report are subject to at least four limitations. First, persons who participated in this investigation might be systematically different from those who refused or were not eligible for the study, and therefore, might not be representative of the U.S. population. Second, matching was not maintained in this analysis because some potential participants contacted declined to participate in the interview or were ineligible. However, this was accounted for in the analytic approach. Third, unmeasured confounding is possible because different types of telework options were not operationalized, nor were participants asked whether their employer provided a specific alternative work site policy. The question assessing telework status did not differentiate work and school settings, however only eight participants in the sample reported being a student. Further, case-patients and control-participants received testing at outpatient testing centers and cannot be generalized to represent serious illness or persons who used other testing modalities. Finally, symptomatic adults with negative SARS-CoV-2 test results might have been infected with other respiratory viruses and case or control status might be subject to misclassification due to limitations of PCR-based testing (*8*,*9*).

Allowing and encouraging the option to work from home or telework, when possible, is an important consideration for reducing SARS-CoV-2 transmission. Characterizing work from home experiences as well as exploring workplace exposures alongside other community exposures will be critical to understanding the impact of mitigation efforts on COVID-19 incidence. Businesses and employers should promote alternative work site options, such as telework, to support worker and community safety during the COVID-19 pandemic. Within the critical infrastructure and other workplaces where telework options are not possible, worker safety measures should continue to be scaled up by creating a COVID-19 preparedness response plan, implementing essential infection prevention and control measures (e.g., social distancing, wearing masks, provision of personal protective equipment, daily health checks, hand hygiene, sanitation, and disinfection), as well as enhancing policies to protect employees and the community.^¶¶^

**Summary**

**What is already known about this topic?**

Since March 2020, large scale measures to reduce workplace transmission of SARS-CoV-2, including workplace closures and providing telework options, have been implemented.

**What is added by this report?**

Adults who received positive test results for SARS-CoV-2 infection were more likely to report exclusively going to an office or school setting in the 2 weeks before illness onset, compared with those who tested negative, even among those working in a profession outside of the critical infrastructure.

**What are the implications for public health practice?**

Businesses and employers should promote alternative work site options, such as teleworking, where possible, to reduce exposures to SARS-CoV-2. Where telework options are not feasible, worker safety measures should continue to be scaled up to reduce possible worksite exposures.

## Footnotes

* https://www.cdc.gov/coronavirus/2019-ncov/community/guidance-business-response.html.

† Response options for critical infrastructure jobs included “Healthcare facility (not in a long-term care facility), healthcare facility (long term care facility), large factory setting, correctional or detention facility, and teacher, educator, or camp counselor (i.e., those who worked with persons aged <18 years).”

§ Baystate Medical Center, Springfield, Massachusetts; Beth Israel Deaconess Medical Center, Boston, Massachusetts; University of Colorado School of Medicine, Aurora, Colorado; Hennepin County Medical Center, Minneapolis, Minnesota; Intermountain Healthcare, Salt Lake City, Utah; Ohio State University Wexner Medical Center, Columbus, Ohio; Wake Forest University Baptist Medical Center, Winston-Salem, North Carolina; Vanderbilt University Medical Center, Nashville, Tennessee; Johns Hopkins Hospital, Baltimore, Maryland; Stanford University Medical Center, Palo Alto, California; University of Washington Medical Center, Seattle, Washington. Participating states include California, Colorado, Maryland, Massachusetts, Minnesota, North Carolina, Ohio, Tennessee, Utah, and Washington.

¶ Other languages included Arabic, Portuguese, Russian, Spanish, and Vietnamese.

** 45 C.F.R. part 46, 21 C.F.R. part 56; 42 U.S.C. Sect. 241(d); 5 U.S.C. Sect. 552a; 44 U.S.C. Sect. 3501 et seq.

†† Cardiac condition, hypertension, asthma, chronic obstructive pulmonary disease, immunodeficiency, psychiatric condition, diabetes, and obesity.

§§ Response options for telework status included “going into an office/school regularly,” “working from home/teleworking,” or “both.” Persons who reported “both” (33) were included among persons who worked from home or teleworked at least part of the time.

¶¶ https://www.cdc.gov/coronavirus/2019-ncov/community/worker-safety-support/index.html; https://www.osha.gov/Publications/OSHA3990.pdf.
